# Cellular Expression of Smarca4 (Brg1)-regulated Genes in Zebrafish Retinas

**DOI:** 10.1186/1471-213X-11-45

**Published:** 2011-07-14

**Authors:** Monica R Hensley, Farida Emran, Sylvia Bonilla, Liyun Zhang, Wenxuan Zhong, Paul Grosu, John E Dowling, Yuk Fai Leung

**Affiliations:** 1 Department of Biological Sciences, Purdue University, 915 W. State Street, West Lafayette, Indiana, 47907, USA; 2 Department of Molecular and Cellular Biology, Harvard University, 16 Divinity Avenue, Cambridge, MA, 02138, USA; 3 Department of Statistics, University of Illinois at Urbana-Champaign, 725 S. Wright Street, Champaign, IL 61820, USA; 4 Center for Genomics Research, Harvard University, 7 Divinity Avenue, Cambridge, MA, 02138, USA

## Abstract

**Background:**

In a recent genomic study, Leung et al. used a factorial microarray analysis to identify Smarca4 (Brg1)-regulated genes in micro-dissected zebrafish retinas. Two hundred and fifty nine genes were grouped in three-way ANOVA models which carried the most specific retinal change. To validate the microarray results and to elucidate cellular expression patterns of the significant genes for further characterization, 32 known genes were randomly selected from this group. *In situ *hybridization of these genes was performed on the same types of samples (wild-type (WT) and *smarca4^a50/a50 ^*(*yng*) mutant) at the same stages (36 and 52 hours post-fertilization (hpf)) as in the microarray study.

**Results:**

Thirty out of 32 riboprobes showed a positive *in situ *staining signal. Twenty seven out of these 30 genes were originally further classified as Smarca4-regulated retinal genes, while the remaining three as retinal-specific expression independent of Smarca4 regulation. It was found that 90.32% of the significant microarray comparisons that were used to identify Smarca4-regulated retinal genes had a corresponding qualitative expression change in the *in situ *hybridization comparisons. This is highly concordant with the theoretical true discovery rate of 95%. Hierarchical clustering was used to investigate the similarity of the cellular expression patterns of 25 out of the 27 Smarca4-regulated retinal genes that had a sufficiently high expression signal for an unambiguous identification of retinal expression domains. Three broad groups of expression pattern were identified; including 1) photoreceptor layer/outer nuclear layer specific expression at 52 hpf, 2) ganglion cell layer (GCL) and/or inner nuclear layer (INL) specific expression at both 36 & 52 hpf, and 3) GCL and/or INL specific expression at 52 hpf only. Some of these genes have recently been demonstrated to play key roles in retinal cell-type specification, differentiation and lamination. For the remaining three retinal-specific genes that are independent of Smarca4 regulation, they all had a subtle expression difference between WT and *smarca4^a50/a50 ^*retinas as detected by *in situ *hybridization. This subtle expression difference was also detected by the original microarray analysis. However, the difference was lower than the fold change cut-off used in that study and hence these genes were not inferred as Smarca4-regulated retinal genes.

**Conclusions:**

This study has successfully investigated the expression pattern of 32 genes identified from the original factorial microarray analysis. The results have demonstrated that the true discovery rate for identifying Smarca4-regulated retinal genes is 90.3%. Hence, the significant genes from the microarray study are good candidates for cell-type specific markers and will aid further investigation of retinal differentiation.

## Background

The retina contains six neuronal cell types and one major glial cell type, all of which originated from the same progenitor cell. In vertebrate retinas, the early proliferation of multipotent progenitors produces a sufficient number of cells that will be specified as different retinal cell types at around the time of their cell cycle withdrawal. The cell types that can be specified at a particular stage of development are controlled by both intrinsic and extrinsic signals; and this temporal restriction on cell fate specification is commonly referred to as the competence model [1]. In general, ganglion cells (GCs) are the first cell type while Muller cells (MCs) are the last cell type to be specified. These reversibly specified cells then undergo irreversible determination after which their cell fate can not be changed by external signals [2]. Specification and determination are collectively called commitment. Finally, these committed cells undergo terminal differentiation during which they send out neuronal processes and synapse with each other.

Many intrinsic factors and extrinsic signals that control retinal cell specification and differentiation have been identified. For example, Atoh7 and Crx are transcription factors that specify retinal ganglion cells [3] and photoreceptors [4,5] respectively. Research on zebrafish retina has also identified key signalling molecules and processes that regulate terminal differentiation and lamination. These include Shh [6], cell polarity regulation [7], cell adhesion [8] and chromatin remodelling [9]. For example, in a retinal terminal differentiation mutant *smarca4^a50/a50 ^*(*young/yng*), all retinal cell types could be specified but failed to fully differentiate due to a null mutation of a chromatin remodelling component *smarca4 *(*brg1*) [9,10]. Nonetheless, the underlying genetic circuitry that controls terminal differentiation is still poorly understood [11].

In a recent microarray study of retinal development in zebrafish, a factorial design was used to identify specifically Smarca4-regulated retinal genes in micro-dissected wild-type (WT) and *smarca4^a50/a50 ^*retinas [12]. Seven hundred and thirty one genes were identified as regulated by Smarca4 with different levels of retinal specificity, as defined by the factorial ANOVA model used. In particular, 259 genes were grouped in the three-way ANOVA models and considered to have the most specific retinal change. To study the regulation of these genes during retinal development, it is essential to validate their differential expression and elucidate their cellular expression patterns. The present study investigated the cellular expression patterns of 32 randomly selected known genes from this group of three-way ANOVA models by whole-mount *in situ *hybridization. A comparative analysis of the results with the original microarray findings was performed to evaluate the performance of the factorial microarray analysis.

## Results

### *In situ *hybridization analysis of Smarca4-regulated genes validates differential expression results obtained by factorial microarray analysis

In a recent factorial microarray analysis that aimed at identifying Smarca4-regulated retinal genes in micro-dissected retinas [12], 259 genes were categorized in three-way ANOVA models (see Methods for details). These genes have the most specific gene expression change in the *smarca4^a50/a50 ^*retinas at 52 hours post-fertilization (hpf). Since the differentiating retinal cells are beginning to organize themselves into three cellular layers and synapse with each other at this stage, the cellular expression analysis of these Smarca4-regulated retinal genes would potentially assist further functional characterization of retinal differentiation and lamination.

To facilitate performance evaluation of this microarray analysis approach, 32 known genes were randomly selected from these 259 genes and whole-mount *in situ *hybridization performed on embryos collected at the same stages that were used in the microarray analysis. These include WT and *smarca4^a50/a50 ^*embryos at 36 & 52 hpf. The corresponding retinal samples at the same stages are WR36 & WR52 for WT, and YR36 & YR52 for *smarca4^a50/a50 ^*respectively (former nomenclature *yng *will be used in the sample abbreviations for *smarca4^a50/a50 ^*to aid comparison with the published data in [12]). The factorial microarray analysis also further categorized these 259 genes into three functional groups: (i) Smarca4-regulated retinal differentiation genes (194 genes), (ii) retinal-specific genes independent of Smarca4 regulation (35 genes), and (iii) Smarca4-regulated genes outside retina (54 genes). Only genes in the first group have a significant differential retinal gene expression in *smarca4^a50/a50 ^*compared to WT (see Methods for the statistical criteria).

Among the 32 randomly selected known genes for *in situ *hybridization, 29 (*olfm2, rlbp1l, guk1, ndrg1, glra4b, robo2, irx7, barhl2, vangl1, dtnbp1, lmo4l, cdh11, elovl4, ctbp2, irx4a, rho, id2a, sv2b, wnt11, calb2l, ckmt1, kal1a, aanat2, pbx1a, fzd8b, tfap2a, nme2l, rcv1 *and *gnat1*) are in the functional group (i) as described above, three (*foxn4, six3a *and *bhlhe22*) are in group (ii) and none is in group (iii). Two (*cdh11 *and *wnt11*) out of the 29 genes from group (i) showed no discernable signal in all retinal samples in the *in situ *hybridization experiments. For *cdh11*, there was considerable expression signal detected outside the eye region (data not shown). This indicates that the probe could detect gene expression. However, there was no discernible expression signal in the eye. For *wnt11*, since there was no signal in the whole embryo (data not shown), an additional probe that is deposited in the public domain (ZFIN ID: ZDB-GENE-990603-12; probe cb748) was acquired and the *in situ *hybridization repeated with *smarca4^a50/a50 ^*and their WT siblings, as well as WT embryos collected from AB and TL strains. In all conditions, the staining in the other reported embryonic regions, including otic vesicles, lower jaw and myoseptum, was clearly observed. However, there was no positive staining in the retinas, even after an overnight staining (Additional file 1, Figure S1 and data not shown). Thus, the *in situ *hybridization results do not support the expression of *wnt11 *and *cdh11 *in the retinas at 36 and 52 hpf.

**Figure 1:**
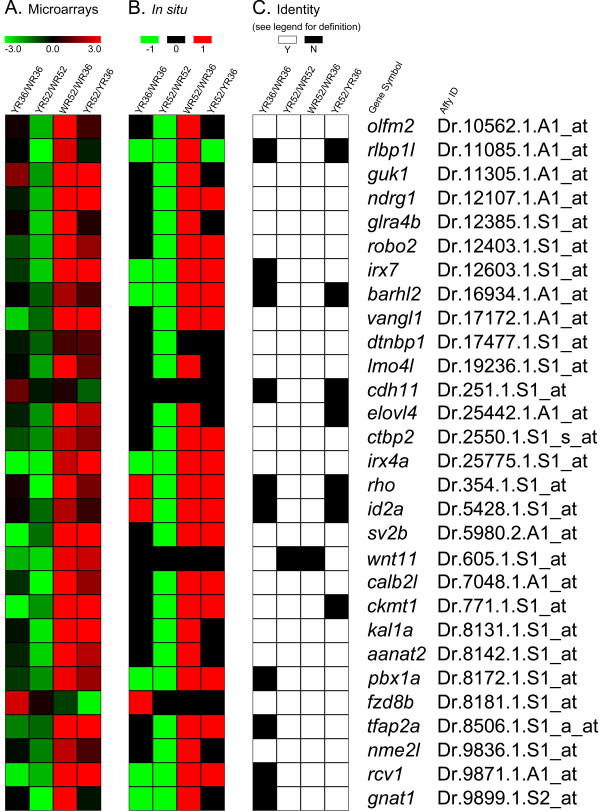
**A comparison of the microarray results with the corresponding *in situ *hybridization experiments**. (A) A heatmap of the fold changes of all retinal comparisons obtained by the original microarray analysis [12](Additional file 2, Table S1). The corresponding gene symbols and AffyIDs are shown on the right. All fold changes are plotted on the log_2 _scale. Red colour indicates an over-expression in that comparison, green indicates an under-expression and black indicates no change. YR36 and YR52: *smarca4^a50/a50 ^*retinal samples at 36 and 52 hpf respectively; WR36 and WR52: WT retinal samples at 36 and 52 hpf respectively. Note that fold change was only one of the criteria for significance inference in the original study; see (C) and Methods for details. (B) A heatmap of the corresponding retinal comparisons in the *in situ *hybridization samples as in (A). Even though all samples for the same gene were stained for the same time, it was decided the best usage of the *in situ *hybridization results was for a qualitative evaluation of differential expression. An over-expression was represented as red (1), no change as black (0) and under-expression as green (-1). (C) An identity matrix of the microarray results compared with the *in situ *hybridization results. An identical observation between the two studies is defined as either both the microarray comparison was significant and the *in situ *hybridization comparison showed a corresponding differential expression, or both the microarray comparison was insignificant and the corresponding *in situ *hybridization did not show a differential expression. A significant microarray result is defined as the specific contrast for that comparison was significant and the corresponding fold change is > = 2 (See Methods and [12]). White colour indicates that the expressions are identical (Y: yes) between microarray and *in situ *hybridization while black indicates otherwise (N: no).

The positive and negative expression results obtained by *in situ *hybridization serve two purposes. First, they can be used to validate the expression changes obtained by the microarray analysis and in turn the significance inference process. This helps evaluate the performance of the approach. Second, the cellular expression patterns of these candidate genes may shed light on their functions. To validate the expression changes obtained by the microarray analysis, the fold changes of the 29 Smarca4-regulated retinal genes from group (i) in four comparisons of retinas (YR36/WR36, YR52/WR52, WR52/WR36 and YR52/YR36) were first plotted in a heatmap (Figure 1A; the fold changes can be found in Additional file 2, Table S1). These data were then compared with the corresponding expression data obtained by *in situ *hybridization. Even though all samples for each gene were stained for the same period of time, since whole-mount *in situ *hybridization is not a true quantitative analysis, it was decided that the best way to interpret the data was to elucidate whether the expression level was higher (1), lower (-1) or not changed (0) in the corresponding comparisons (Figure 1B; Additional file 2, Table S1). First, the expression changes in the *in situ *hybridization were highly similar to the corresponding fold changes in the microarray analysis (compare Figure 1A &1B). Furthermore, an identity matrix was established to compare the observations for all four retinal comparisons (YR36/WR36, YR52/WR52, WR52/WR36 and YR52/YR36) obtained from the microarray analysis with the *in situ *hybridization (Figure 1C). It can be shown that in 83.6% (97/116 comparisons) of the cases, when there was a significant change in the microarray comparison (see Methods for a detailed definition of statistical significance); there was a noticeable corresponding *in situ *hybridization expression change or vice versa (Figure 1C). Finally, since these genes were originally selected by statistical criteria focused on the *smarca4^a50/a50 ^*vs. WT comparisons, it can be further demonstrated that the identical rate of having a corresponding expression change in the *in situ *hybridization with only these significant microarray comparisons is 90.3% (28 out of 31 comparisons; Additional file 2, Table S1A, bold-type observations in columns 1 & 2 for microarray comparisons). To see if these true positive observations and the theoretical true discovery rate (>95%) for significance inference in the microarrays are concordant, a z test was conducted. The *p*-value is 0.83, suggesting that there was no significant difference between the theoretical true discovery rate [12] and the actual observation. Together, these results suggest that the original factorial microarray analysis can effectively identify true differential expression in micro-dissected zebrafish retinas.

### Cellular expression patterns of Smarca4-regulated retinal genes

**Figure 2:**
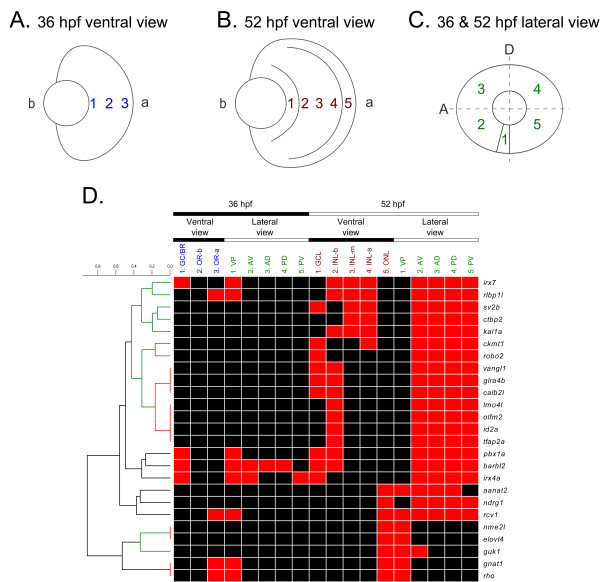
**Hierarchical clustering of the cellular expression patterns of 25 Smarca4-regulated retinal genes in WT retinas**. Twenty five out of the 27 Smarca4-regulated retinal genes had a signal intensity that was sufficiently high for discerning their normal cellular expression domains in WT retinas at 36 and 52 hpf unambiguously. The retinas at these stages were artificially divided into several domains (A, B & C) and positive expression in that region scored. The resulting observations are plotted as a heatmap, with red colour representing a positive signal in a particular retinal domain and black representing no discernible signal. The columns are arranged according to the expression domains as defined in (A, B & C). A hierarchical clustering was conducted to elucidate the similarity of the cellular expression patterns of these genes (D). The resulting dendrogram of the clustering is plotted on the left side of the heatmap and the rows of the heatmap are arranged accordingly. The highly significant clades of the dendrogram are shown in red (*p-*value < 0.05) and green (*p-*value < 0.1).

The cellular expression patterns of the 27 Smarca4-regulated genes with positive *in situ *hybridization signals in the WT retinas were further analyzed as follows. First, their expression domains in 36 and 52 hpf retinas were recorded from both ventral and lateral views, defined as follows (Additional file 3, Table S2): (A) For the 36 hpf ventral view, three expression domains were defined: 1. ganglion cell/basal region (GC/BR), 2. outer retina-basal (OR-b) and 3. outer retina-apical (OR-a)(Figure 2A). The basal region should contain differentiating ganglion cells at this stage [13]; however since ganglion cell marker staining were not performed in this study in conjunction with the *in situ *hybridization, this region may contain cells that belonged to other cell types. Thus it is more appropriate to define the expression domain as GC/BR. The remaining part of the retina has not yet differentiated at this stage and was defined as OR-b and OR-a to reflect the observed difference in expression pattern within this area. (B) For the 52 hpf ventral view, five expression regions were defined: 1. ganglion cell layer (GCL), 2. inner nuclear layer-basal (INL-b), 3. inner nuclear layer-middle (INL-m), 4. inner nuclear layer-apical (INL-a) and 5. outer nuclear layer (ONL)(Figure 2B). Several cell types will ultimately differentiate in the INL, including amacrine cells (ACs), bipolar cells (BCs), horizontal cells (HCs) and MCs, while photoreceptors will occupy the ONL. (C) For the lateral view for both 36 and 52 hpf, five expression domains were defined: 1. ventral patch (VP), 2. anterior-ventral (AV), 3. anterior-dorsal (AD), 4. posterior-dorsal (PD) and 5. posterior-ventral (PV)(Figure 2C). The VP has been used to refer to the location from which cells in GCL, INL and ONL first differentiate [14,15]; and is used here to generally refer to staining domain that is found in a very restricted manner on the ventral side of the retina.

**Figure 3:**
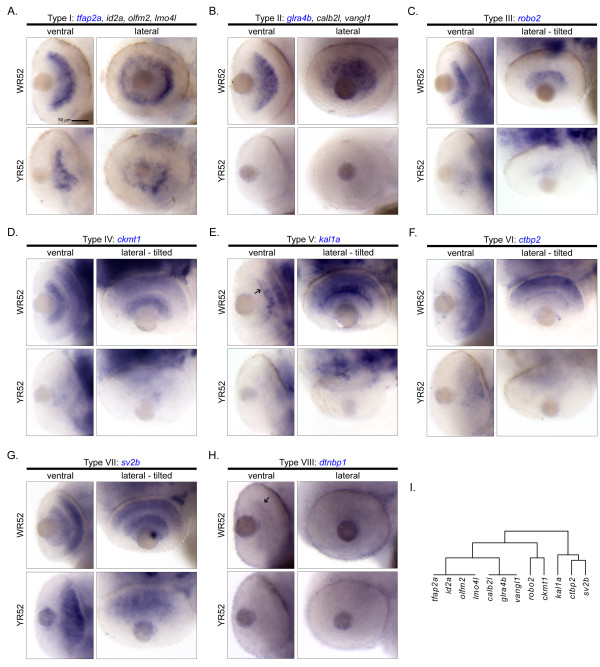
**Cellular expression patterns of Smarca4-regulated retinal genes specifically expressed in GCL and/or INL at 52 hpf only**. A total of 14 genes were clustered in a clade with a relatively significant *p-*value (< 0.1; Figure 2). Except for *rlbp1l *and *irx7 *that expressed at both 36 and 52 hpf and are shown in Figure 5, all remaining 12 genes only expressed at 52 hpf in GCL and/or INL. There are eight types of cellular expression pattern: (A) *tfap2a, id2a, olfm2 *&* lmo4l*, (B) *glra4b, calb2l *&* vangl1*, (C) *robo2*, (D) *ckmt1*, (E) *kal1a*, (F) *ctbp2*, (G) *sv2b *and (H) *dtnbp1*. If more than one gene has the same expression pattern, only one example is shown and its name is highlighted in blue. For each gene, the ventral and the lateral views of WT and *smarca4^a50/a50 ^*retinas are shown. In some cases, the embryo was slightly tilted from the lateral view to facilitate a better observation of the expression domain. Some specific expression locations in the retina are highlighted by black arrows. The corresponding clade of the dendrogram from Figure 2D is reproduced in (I). Refer to Figure 1 for sample abbreviations. Scale bar: 50 μm.

**Figure 4:**
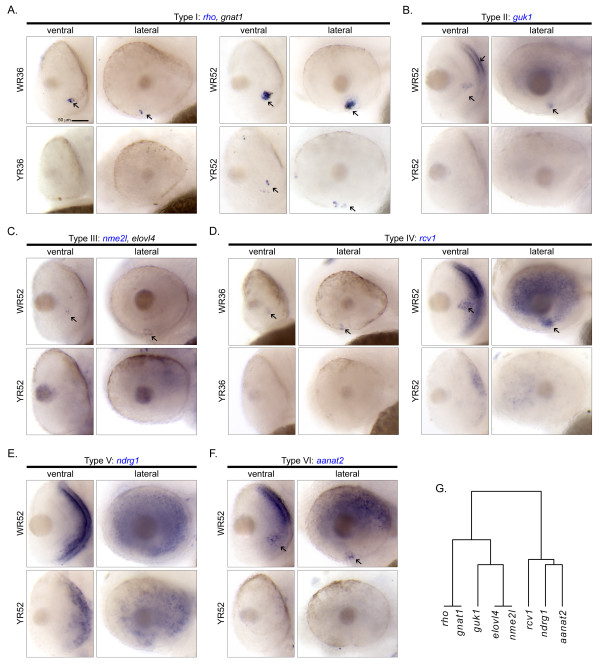
**Cellular expression patterns of Smarca4-regulated retinal genes expressed specifically in photoreceptor layer/ONL at 52 hpf**. A total of eight genes were found to express in the photoreceptor layer/ONL. There are six types of cellular expression pattern: (A) *rho *&* gnat1*, (B) *guk1*, (C) *nme2l *&* elovl4*, (D) *rcv1*, (E) *ndrg1 *and (F) *annat2*. If more than one gene has the same expression pattern, only one example is shown and its name highlighted in blue. For each gene, the ventral and the lateral views of WT and *smarca4^a50/a50 ^*retinas are shown. Some specific expression locations in the retina are highlighted by black arrows. The corresponding clade of the dendrogram from Figure 2D is reproduced in (G). Refer to Figure 1 for sample abbreviations. Scale bar: 50 μm.

**Figure 5:**
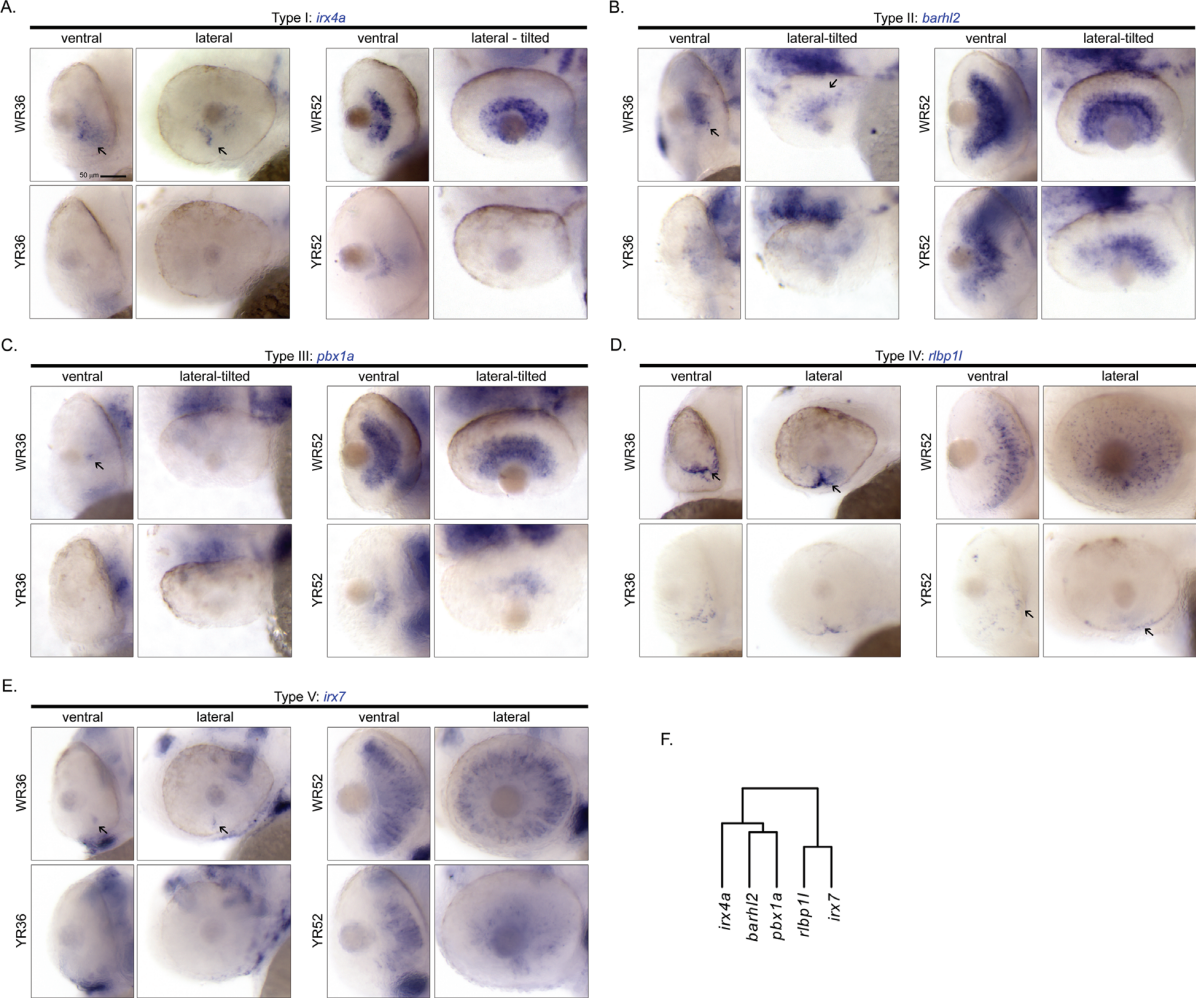
**Cellular expression patterns of Smarca4-regulated retinal genes specifically expressed in GCL and/or INL at both 36 and 52 hpf**. A total of five genes: (A) *irx4a*, (B) *barhl2*, (C) *pbx1a*, (D) *rlbp1l *and (E) *irx7 *were found to express at both 36 and 52 hpf in GCL and/or INL in the WT retinas. Each one of them has a unique cellular expression pattern. For each gene, the ventral and the lateral views of WT and *smarca4^a50/a50 ^*retinas are shown. In some cases, the embryo was slightly tilted from the lateral view to facilitate a better observation of the expression domain. Some specific expression locations in the retina are highlighted by black arrows. The corresponding clade of the dendrogram from Figure 2D is reproduced in (F). Refer to Figure 1 for sample abbreviations. Scale bar: 50 μm.

Then, the similarity of the cellular expression domains from these views between different genes was investigated by hierarchical clustering (Figure 2D). *Dtnbp1 *and *fzd8b *were excluded from this clustering analysis. This is because *dtnbp1*'s expression was only intense enough for discerning an over-expression in WT compared with *smarca4^a50/a50 ^*retinas, but not for an unambiguous elucidation of the cellular expression domains (Figure 3H). While for *fzd8b*, it was only over-expressed in YR36 and not in either of the WT conditions; thus it would not be informative to include this gene in the clustering of WT cellular expression patterns (see sub-section IV below). The clustering analysis using the remaining 25 genes revealed three general groups of expression patterns: 1) photoreceptor layer/ONL specific expression at 52 hpf (Figure 4), 2) GCL and/or INL specific expression at both 36 & 52 hpf (Figure 5) and 3) GCL and/or INL specific expression at 52 hpf only (Figure 3).

### I. Photoreceptor layer/ONL specific expression at 52 hpf

A total of eight genes, including *rho, gnat1, guk1, nme2l, elovl4, rcv1, ndrg1 *and *aanat2*, were found to express specifically in photoreceptor layer/ONL in the WT retinas (Figure 4). Their expression was suppressed in all *smarca4^a50/a50 ^*retinas. Two sub-clusters were also observed (Figure 4G). The genes in the first sub-cluster only expressed in a ventral patch of cells in the retina by 52 hpf (*rho *&*gnat1*, Figure 4A; *nme2l *&*elovl4*, Figure 4C). The genes in the second sub-cluster expressed to a different degree in the other parts of the ONL at the same stage in addition to the ventral patch of the retina (*guk1*, Figure 4B; *rcv1 *and *aanat2*, Figure 4D &4F respectively), except for *ndrg1 *which did not express in this domain (Figure 4E). Also, the expression patterns of *rho *and *gnat1*, as well as *nme2l *and *elovl4 *were highly similar and were tightly clustered with each other (*p-*value <0.05; Figure 2D, red colour in the dendrogram). Interestingly, *rho, gnat1 *and *rcv1*, components of the visual cycle, were also weakly expressed in the ventral retina at 36 hpf (Figure 4A &4D). This is earlier than the stage (~50-52 hpf) of the first *rho *expression previously reported [16], but is consistent with the public *in situ *hybridization data (ZFIN ID: ZDB-GENE-990415-271), which show the first expression of *rho *in the same location at around the same stage (Prim-15 - 25; ~30-36 hpf).

### II. GCL and/or INL specific expression at both 36 & 52 hpf

A group of five genes, including *irx4a, barhl2, pbx1a, rlbp1l *and *irx7*, were expressed in WT retinas at both 36 and 52 hpf (Figure 2D). Their expressions were all suppressed in *smarca4^a50/a50 ^*retinas at the corresponding stages. These genes were segregated into two sub-clusters because of their diverse expression patterns at 36 hpf (Figure 5F). The first sub-cluster contains *irx4a, barhl2 *and *pbx1a *(Figure 5A, B &5C). This group of genes expressed in the GC region at 36 hpf to a variable degree (Figure 5, arrows). For example, *barhl2 *was expressed in the VP, AV, AD and PD but not in the PV domain from the ventral view (Figure 5B, arrows), suggesting that it is a gene that may be regulated by the neurogenic wave in the retina [13]. By 52 hpf, all of these three genes were expressing in either the GCL or both GCL and INL-b, which contains the presumptive ACs. The second sub-cluster, containing *rlbp1l *and *irx7 *(Figure 5D &5E), is clustered within a larger family of genes that only expressed at 52 hpf and will be discussed in the next section. Nonetheless, these two genes have a slightly unique expression pattern: whereas they were expressed on the ventral side of the 36 hpf retinas (Figure 5D &5E, arrows), their expression domain was restricted to the whole INL by 52 hpf.

### III. GCL and/or INL specific expression at 52 hpf only

A total of 14 genes (*irx7, rlbp1l, sv2b, ctbp2, kal1a, ckmt1, robo2, vangl1, glra4b, calb2l, lmo4l, olfm2, id2a *and *tfap2a*) were clustered in a group with a *p-*value < 0.1 (Figure 2D, green colour part of the dendrogram). Among them, only *irx7 *and *rlbp1l *started to express at 36 hpf and were discussed above (Figure 5D &5E). For the remaining 12 genes in the cluster, they only expressed at 52 hpf (Figure 3). Interestingly, several genes inside this cluster have a highly similar and unique expression pattern in that they were tightly grouped in two sub-clusters with a *p-*value* *< 0.05 (Figure 2D, red colour part of the dendrogram). The first sub-cluster consists of four genes - *tfap2a, id2a, olfm2 *and *lmo4l *(Figure 3A) that exclusively expressed in the INL-b, and three genes - *calb2l, glra4b *and *vangl1 *(Figure 3B) that expressed in both GCL and INL-b. The second sub-cluster consists of two genes - *robo2 *(Figure 3C) and *ckmt1 *(Figure 3D) that both expressed strongly in GCL. Also, *ckmt1 *had an addition expression domain in the INL-a. There were three genes - *kal1a *(Figure 3E), *ctbp2 *(Figure 3F) and *sv2b *(Figure 3G) that did not belong to these two sub-clusters and formed a separate one. All of them had broad expression domains in INL that at least spanned the INL-m and INL-a. *Sv2b *further expressed in the GCL as well. Interestingly, *kal1a *was strongly expressed in a subset of cells in the AC region that the neuronal processes were intensely stained and highlighted the inner plexiform layer (IPL) (Figure 3E, arrow).

### IV. *Fzd8b *only expressed in *smarca4^a50/a50 ^*retinas at 36 hpf

**Figure 6:**
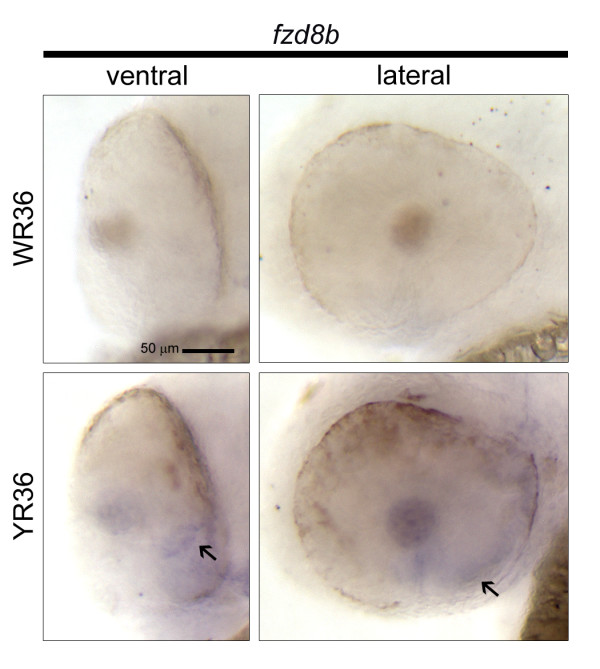
**Cellular expression pattern of *fzd8b *which only expressed in *smarca4^a50/a50 ^*retinas at 36 hpf**. *Fzd8b *is the only gene in the current study whose detectable expression was in the *smarca4^a50/a50 ^*retinas at 36 hpf. The ventral and the lateral views of these retinas are shown. The specific expression domains in the *smarca4^a50/a50 ^*retinas are highlighted by black arrows. Refer to Figure 1 for sample abbreviations. Scale bar: 50 μm.

*Fzd8b *is a gene that only expressed in *smarca4^a50/a50 ^*retinas at 36 hpf at a very low level. Its expression appeared to be restricted to the PV and the choroid fissure area (Figure 6). Interestingly, it was not expressed at a discernible level in other tissues and stages in both the microarray and *in situ *hybridization experiments. This suggests that there was a specific up-regulation of *fzd8b *in the *smarca4^a50/a50 ^*retinas at 36 hpf due to the lack of a functional Smarca4.

### Cellular expression patterns of retinal-specific genes that were not inferred to be regulated by Smarca4

**Figure 7:**
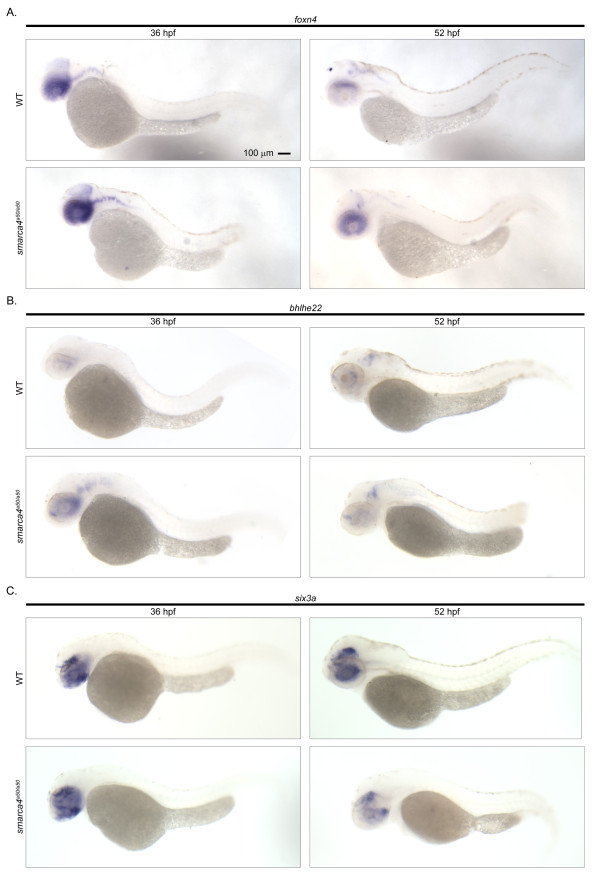
**Cellular expression patterns of retinal-specific genes that were not inferred to be Smarca4-regulated**. A total of three genes: (A) *foxn4*, (B) *bhlhe22 *and (C) *six3a *were not inferred by the original microarray analysis as Smarca4-regulated genes but were inferred to have a retinal-specific expression. The lateral view of a whole WT and *smarca4^a50/a50 ^*embryo at 36 and 52 hpf is shown to illustrate the retinal specific expression. Note that there were subtle differential expression changes in *smarca4^a50/a50 ^*retinas, but they did not meet the fold change cut-off criterion used in the microarray significance inference. See text for details. Scale bar: 100 μm.

*Foxn4, bhlhe22 *and *six3a *did not meet the original microarray analysis criteria to be Smarca4-regulated retinal genes (See Methods for details) and were classified in the functional group (ii) that were shown to have a retinal specific expression at both 36 and 52 hpf (Additional file 4, Table S3). The *in situ *hybridization analysis confirmed this retinal specific expression compared to the whole body at all stages (Figure 7A, B &7C). However, it was also noticed that there was a discernible expression difference between WT and *smarca4^a50/a50 ^*retinas in the *in situ *samples in several instances. These include a slight increase of *foxn4 *(Figure 7A) in *smarca4^a50/a50 ^*retinas at 36 & 52 hpf, a slight increase and decrease of *bhlhe22 *in *smarca4^a50/a50 ^*retinas at 36 and 52 hpf respectively (Figure 7B), and a slight decrease of *six3a *in *smarca4^a50/a50 ^*retinas at 52 hpf (Figure 7C). An inspection of the original microarray results indicates that the sign of the fold changes matched with the expression changes as determined by *in situ *hybridization and the average microarray expression values in all these comparisons were considerably higher than the background. However, the magnitude of the fold changes was not larger than 2 (*foxn4: *YR36/WR36 = 1.06, YR52/WR52 = 1.73; *bhlhe22*: YR36/WR36 = 1.35, YR52/WR52 = - 1.17; *six3a*: YR36/WR36 = 1.34, YR52/WR52 = -1.29). This suggests that the selection criteria were stringent in inferring Smarca4-regulated retinal genes, but might be too stringent for detecting subtle changes. Alternatively, *in situ *hybridization, despite being able to reveal cellular expression patterns, is intrinsically not a quantitative method. The detection threshold varies and is affected by a number of experimental factors. The changes as observed may not be superior to the microarrays measurements. Hence it would be wise to combine both types of approach and stringent statistical criteria for selecting candidates for further experimental investigations.

## Discussion

This study has successfully investigated the expression of 32 genes identified from a microarray study of Smarca4-regulated retinal genes from micro-dissected zebrafish retinas and elucidated the cellular expression patterns. The original microarray analysis has inferred 29 of these 32 genes as Smarca4-regulated retinal genes and the remaining three as retinal specific genes whose expression is independent of Smarca4 regulation. The *in situ *hybridization analysis has demonstrated that the sign of the expression changes matches with the microarray results well (Figure 1). In particular, 90.3% (28/31 cases) of the significant microarray expression comparisons have a corresponding differential expression between WT and *smarca4^a50/a50 ^*retinas in the *in situ *hybridization experiments. This suggests strongly that factorial microarray analysis, can efficiently detect true biological differences among different types of micro-dissected zebrafish retinas. Twenty seven out of the 29 Smarca4-regulated retinal genes have a positive *in situ *hybridization signal in the retinas, and 25 of them have the cellular expression domains that could be clearly discerned. Three general groups of expression patterns can be identified from these 25 genes (Figure 2D): 1) photoreceptor layer/ONL specific expression at 52 hpf, 2) GCL and/or INL specific expression at both 36 & 52 hpf and 3) GCL and/or INL specific expression at 52 hpf only.

The acquisition of the cellular expression patterns and the clustering of these genes can facilitate downstream characterization, because genes that are expressed in the same domain are likely to be regulated by the same upstream factor(s) and/or are used to control development and functions of specific cell type(s). For example, a group of photoreceptor/ONL genes were expressed on the ventral side of WT retinas at 52 hpf and were suppressed in the *smarca4^a50/a50 ^*retinas (Figure 4). This is the beginning stage of photoreceptor generation in the ventronasal patch of the retina [14], and many of these genes are related to the visual cycle. Interestingly, *rcv1 *and *aanat2 *are expressed in virtually the whole ONL at 52 hpf in addition to the ventronasal patch (Figure 4D &4F), while *ndrg1 *is expressed in the whole ONL but not the ventronasal patch at this stage (Figure 4E). This extensive expression pattern is obviously different from the previously reported expression patterns of cones and rods differentiation, as indicated by the wave-like expression pattern of cone opsin and sporadic expression of rod opsin respectively starting from the ventronasal retina at around 52 hpf [16]. Since *rcv1 *also participates in the visual cycle [17], the extensive expression of *rcv1, aanat2 *and *ndrg1 *in the ONL at 52 hpf suggests that there may be a different regulatory circuit that controls their expression. Together with the detection of *rho, gnat1 *and *rcv1 *in the ventral retina at 36 hpf (Figure 4A &4D), these observations also suggest that the differentiation program of photoreceptors may begin earlier than 50-52 hpf. In the second example, a few genes including *tfap2a, id2a, olfm2 *and *lmo4l *(Figure 3A), are expressed specifically on the basal side of the INL which is the presumptive region of ACs. Their expression domains were highly overlapped with each other that these genes were grouped together in a highly significant sub-cluster (*p-*value < 0.05; Figure 2D). This suggests that they may play a role in ACs development. Indeed, a recent perturbation experiment of *id2a *in zebrafish [18] have demonstrated such a role. In addition, the knockout of an orthologue of *lmo4l*, LMO4, in mice leads to a reduction of amacrine cells [19]. In the third example, *barhl2 *is a gene that has been demonstrated to regulate the development of GCs and ACs in mice [20]. Its expression pattern in the zebrafish correlates nicely with the development of GCs and ACs (Figure 5B), suggesting that *barhl2 *is likely to play a similar role in zebrafish retinal development.

One key objective of the original microarray study was to identify Smarca4-regulated retinal genes because their expression should either mediate terminal differentiation and/or be a consequence of terminal differentiation. The hallmarks of terminal differentiation of neurons include neurite outgrowth and synaptogenesis, which would be manifested as the formation of the retinal lamination. The current *in situ *hybridization analysis of the candidate genes, which are mostly differentially expressed at 52 hpf, during which the retinal lamination is first observed, may potentially provide new insights into the molecular control of this process. There are at least two non-exclusive possibilities: 1) these genes control the differentiation of a specific cell type which in turn orchestrates the formation of the final neuronal connection and lamination and/or 2) these genes control patterning and lamination of the retina directly. In the microarray study, it was speculated that *tfap2, id2 *and *irx *transcription factor families would play a key role in retinal differentiation and lamination [12], and this has been proven by studies of *tfap2a *knockout mice [21], and *id2a *[18] and *irx1a *[22,23] knockdowns in zebrafish. In fact, *id2a *has also been demonstrated to play a role in the development of ACs as discussed above and the knockdown of *tfap2c *in *tfap2a *zebrafish mutant shows a very severe eye development phenotype, in which the eye size becomes significantly smaller [24]. These results together suggest that these transcription factor families are integral components of retinal differentiation and lamination, and that the remaining members of these groups may play key roles in retinal development. Indeed, our investigation has demonstrated that *irx7*, a gene that has an expression pattern that spans the whole INL at 52 hpf, plays a role in the differentiation of both INL and photoreceptors, as well as lamination ([12] & manuscript in prep). Also, in the original microarray study, Cdk5 and activators were demonstrated to play a role in retinal differentiation [12]. The execution of their functions likely involves a subset of the genes presented in this study. Thus, the knockdown of these key mediators of Smarca4-network and subsequent *in situ *hybridization analysis of the other Smarca4-regulated genes, which is currently in progress, will likely further the understanding of retinal differentiation.

While the microarray analysis accurately excluded genes that did not meet all criteria to be Smarca4-regulated retinal genes, some of these excluded genes might have a role in subtle developmental change in retinas that are regulated by Smarca4. For instance, the fold change of a key transcription factor in a network can be less than two, and yet it can play a substantial role in the developmental process that it controls. *Foxn4 *was excluded by the original microarray analysis as being a Smarca4-regulated retinal gene because its fold change was below the two-fold cut-off despite having a significant corresponding contrast. Nonetheless, it was obvious that there was a discernible difference in the expression level and cellular expression pattern of this gene between WR52 and YR52 (Figure 7A). The expression seemed to be relatively higher on the dorsal side of the WR52; while in YR52, the expression was generally higher but there was no such regional difference. In addition, the expression of *foxn4 *at 52 hpf is restricted to the proliferative marginal zone region; while at 36 hpf, it is primarily located in the proliferative part of the retinal neuro-epithelium (Figure 7A and data not shown). These results suggest that *foxn4 *may also play a role in the Smarca4-regulated retinal differentiation through the regulation of the retinal progenitors. Indeed, *foxn4 *has been demonstrated to express in retinal progenitor cells in mice and the null mutant has retinal dysplasia [25]. While a small and consistent fold change of a gene can be biologically significant, it is necessary to implement a cut-off to ensure the false discovery rate is low. This is a necessary trade-off to maximize the number of true positive genes, especially in the first genomic study of micro-dissected zebrafish retinas. It is obvious that while the original factorial microarray analysis have efficiently identified true Smarca4-regulated retinal expression, it is possible that some genes that are subtly regulated by Smarca4 did not meet the statistical cut-off and were excluded from this category.

## Conclusions

This study has investigated the expression of 32 genes identified from a factorial microarray analysis that was designed for searching Smarca4-regulated retinal genes. Twenty nine of them were grouped in this category while three were excluded by the original analysis. The *in situ *hybridization results have strongly supported that the original factorial microarray analysis can efficiently identify true Smarca4-regulated retinal genes with a true positive rate of 90.3%. Thus, this gene list is not only a rich source for cell-type specific markers, but also may assist in formulating new hypothesis to study the underlying genetic regulatory circuits that control retinal development.

## Methods

### Fish

Zebrafish (AB & TL wild-type (WT) and *smarca4^a50/+ ^*(*yng*)[10]) were maintained according to standard procedures [26]. All protocols were approved by the Harvard Standing Committee on the Use of Animals in Research and Teaching and Purdue Animal Care and Use Committee.

### Egg collection, embryo staging and collection

Wild-type (WT) and *smarca4^a50/a50 ^(yng) *embryos were collected at the same stages (i.e. 36 and 52 postfertilization (hpf)) as in the microarray analysis [12]. To ensure all embryos were at a similar stage during collection, embryos were collected every 20 minutes in E3 medium [27]. Zebrafish embryo staging was performed as described [28], with the following modifications. First, embryos from each 20-minute collection were inspected at around 10-12 hpf, and any abnormal looking embryos were discarded. Then a sample of embryos, typically 5-7 for each 20-minute collection, was staged. This would be considered the average stage for all the embryos collected during the same period. The staged embryos were treated with 0.003% phenylthiourea (PTU) (Sigma; St. Louis, MO) in E3 medium starting at 23 hpf to prevent melanization. At least 10 embryos were set aside and kept in E3 as staging reference. The embryos treated with PTU were staged again at 36 and 52 hpf immediately before collection, using the staging reference embryos that were not treated with PTU. Standard staging criteria were used in conjunction with the eye size parameter for these stages [29]. *Smarca4^a50/a50 ^*embryos were staged by using their WT siblings collected from the same clutch at the same time. Embryos were dechorionated by treating with 1 mg/mL Pronase (Sigma; St. Louis, MO) for 1 to 2 minutes until embryos started to come out from the chorion; then all embryos were rinsed extensively with E3 medium. All embryos were immediately fixed in 4% paraformaldhyde (PFA)(Sigma; St. Louis, MO) in phosphate buffered saline (PBS), dehydrated and stored in 100% methanol at -20°C as described [27].

### Complementary DNA library preparation

Total RNA was extracted and purified from 2-day old zebrafish embryos as described [29]. Complementary DNA (cDNA) library was prepared by reverse transcribing messenger RNAs from purified total RNA using Superscript II reverse transcriptase (Invitrogen, Carlsbad, CA) and an anchored primer 5'-dT20VN-3' (Integrated DNA Technologies, Coralville, IA).

### Gene selection for *in situ *hybridization analysis

The factorial design in the original microarray analysis [12] allowed for the modelling of the influence of three biological changes: mutation (*M*; either wild-type or *smarca4^a50/a50 ^*(*yng*)), change in tissue (*R*; either whole embryo or retina) and change in time (*T; *either 36 or 52 hpf) on gene expression level in a particular sample. All genes were first fitted into one of the four most parsimonious ANOVA models: insignificant, one, two or three-way model. In the three-way model group, the expression of a gene *g *(*y_g_*) was modelled as(1)

indicating that the expression of the gene was affected by all three biological changes as well as their two-way and three-way interactions; while in the two-way model group, the expression of a gene *g *(*y_g_*) was modelled as(2)

indicating that the expression of the gene was affected by all three biological changes as well as at least one of their two-way interactions; while in the one-way model group, the expression of a gene *g *(*y_g_*) was modelled as(3)

indicating that the expression of the gene was affected by at least one of the three biological changes but not their interactions; while in the insignificant model group, the expression of a gene *g *(*y_g_*) was modelled as(4)

indicating that the expression of the gene was not affected by any of the three biological changes.

Genes that were categorized in the three-way model group had the most specific regulation in the *smarca4^a50/a50 ^*retinas, because the three-way interaction term (T*M*R) indicates the presence of a mutation-specific effect on gene expression in the retina at 52 hpf. Since this is the stage when retinal lamination is first formed, the first cellular expression investigation was focused on this group of genes. Thirty two known genes were randomly selected from 259 three-way models for further *in situ *hybridization analysis. Also, in the same microarray analysis study, these 259 models were further categorized into three functional groups: (i) Smarca4-regulated retinal genes, (ii) Retina-specific genes independent of Smarca4 regulation, and (iii) Smarca4-regulated genes outside the retina. Note that a significant 3-way interaction term does not necessarily translate to a final overall significance, because that was inferred by two criteria [12]: (1) a significant contrast (false discovery rate *p-*value < 0.05), which is the specific comparison of two biological conditions using the corresponding coefficients from the ANOVA model and (2) fold change of the corresponding comparison > = 2. A comparison can be insignificant because the T*M*R coefficient can cancel out the effect on expression imposed by other terms, even though the T*M*R coefficient is significant by itself. To infer significant Smarca4-regulated retinal genes for the functional group (i), the following contrasts were used:

For YR36/WR36:

H_0_: M + M*R = 0, H_0_: M + M*R = 0, and *H_0_: M = 0 *for Eq. 1, 2 & 3 respectively;

while for YR52/WR52:

H_0_: M + M*R + T*M + T*M*R = 0, H_0_: M + M*R + T*M = 0, and *H_0_: M + T = 0 *for Eq. 1, 2 & 3 respectively.

### Primers Design

The goal of the primers design was to select primers that specifically flanked 500-850 bps of the candidate genes so that the resulting amplicon will overlap with the target sequence of the probesets of the Affymetrix zebrafish whole genome array (Affymetrix, Santa Clara, CA) as much as possible for riboprobe synthesis. Since this GeneChip was used in the microarray study [12], designing a riboprobe with maximal sequence overlap with the probeset sequence also allows for better expression validation of the original microarray results. The primers were designed using the following procedures until a pair of primers was chosen for that particular gene. First, a computer script (available upon request) was written to analyze and locate the consensus sequence of a probeset from the Affymetrix design file. Primers that flanked 500-850 bps including the target region were then selected by Primer3 [30], using default parameters except the option 'Max Poly-X' was set to 3. However, this approach was not always feasible, as Affymetrix often selected the target region for their probesets from the 3' end or the untranslated region (UTR) of a gene. The low complexity of the 3'UTR sometimes precluded an efficient primer design and/or PCR amplification. In this case the target region of the Primer3 would then be moved 5' in 50 bps increments until a pair of primers was picked. Sometime the consensus sequence was too short, i.e. shorter than 500 bps. In that case, the ReqSeq sequence would be obtained by the respective Genbank ID. The longer sequence would then be used for primer design as described above. The specificity of the selected primers was checked by blasting NCBI's zebrafish nr and htgs databases. All primers selected by this exercise are shown in Additional file 5, Table S4.

### Whole-mount *in situ *hybridization

Specific DNA fragments were amplified from a 2-day old zebrafish cDNA library and cloned into pGEM-T easy vector (Promega, Madison, WI) for probe synthesis. All constructs were sequenced verified. Riboprobe synthesis and whole-mount *in situ *hybridization were performed using procedures described [27]. The stringency washes after probe hybridization and before signal detection were performed using the Biolane semi-automated *in situ *hybridization machine (INTAVIS Bioanalytical Instruments AG, Koeln, Germany). The specific parameters of the washing steps for the embryos are listed as follows:

• Twice in 50% formamide/2X SSCT (Saline-Sodium Citrate with 0.1% Tween-20) for 20 minutes each at 65°C

• Once in 2X SSCT for 15 minutes at 65°C

• Twice in 0.2X SSCT for 20 minutes each at 65°C

• Twice in PBST (PBS with 0.1% Tween-20) for five minutes each at room temperature

• Once in Block buffer (2 mg/mL Bovine Serum Albumin, 2% normal sheep serum in PBST) for two hours at room temperature

• Once in 1:3000 Anti-dioxgenin-alkaline phosphatase antibody (Roche Applied Science; Indianapolis, IN) in block for 12 hours at 4°C

◦ The antibody was pre-adsorpted at 1:1000 dilution in Block buffer with at least 400 fixed embryos for an overnight at 4°C before used

◦ The pre-adsoprtion step slightly increases signal clarity, but not very substantial, so this can be considered as an optional preparation

◦ The final working stock can be re-used for a few times and should be stored at 4°C

• Six times in PBST at room temperature; the first one for five minutes, the next four for 30 minutes each and the final one is programmed for 12.5 hours, which can be stopped at any time after the first 30 minutes.

• Three times in staining buffer (100 mM Tris pH9.5, 50mM MgCl2, 100 mM NaCl, 0.1% Tween-20 and 1 mM levamisol (Sigma; St. Louis, MO)) for five minutes each at room temperature

At least 20 embryos were analyzed for each antisense probe for each developmental stage and genotype. The same number of WT embryos at the same developmental stage was also collected and used for the sense probe control. All embryos used for the characterization of the same gene were stained for the same period of time to maximize comparability between conditions. The samples were destained by a 2:1 mixture of benzyl benzoate and benzyl alcohol and stored in 4% PFA at 4°C.

### Imaging

Embryos were mounted in 3% methylcellulose on a depression slide for observation and imaging. All images were taken by a Spot RT3 slider CCD camera (Diagnostic Instruments, Sterling Heights, MI) mounted on an Olympus SZX16 Stereomicroscope (Olympus, Center Valley, PA). The images were subsequently merged using Adobe Photoshop CS3 (Adobe Systems Incorporated, San Jose, CA).

### Data analysis

Hierarchical clustering analysis of the expression domains of the genes in different samples was conducted as follows: First, the expression domain of the genes, as defined in Figure 2A, B &2C, was visually scored as 0 (absent) or 1 (present) (Additional file 3, Table S2). Then the dissimilarity of these expression domains between each gene was calculated by a binary distance measure and hierarchical clustering conducted using average group linkage agglomeration. Finally, *p-*values of the clustering results were calculated by a multi-scale bootstrap resampling method [31]. The number of bootstrap replications was 10000. All data analyses were conducted in the R statistical environment version 2.11.1 http://www.r-project.org. Heatmaps were generated using Multiexperiment Viewer (MeV) http://www.tm4.org.

## Competing interests

The authors declare that they have no competing interests.

## Authors' contributions

MRH coordinated and conducted the *in situ *hybridization experiments and drafted the manuscript. FE participated in the production of the constructs and the *in situ *hybridization experiments. SB and LZ participated in the *in situ *hybridization experiments. WZ participated in data analysis. PG wrote a script for primer selection. JED participated in the interpretation of the experiments and drafted the manuscript. YFL conceived the project, participated in the production of the constructs and the *in situ *hybridization experiments. He also supervised the design, execution and interpretation of the experiments, and drafted the manuscript. All authors have read and approved the final manuscript.

## Supplementary Material

Additional file 1 **Figure S1. Cellular expression pattern of *wnt11 *as identified by a previously reported riboprobe**. The *in situ *hybridization experiment of *wnt11 *was repeated using a probe (cb748) that was obtained from ZIRC. (A) The ventral and the lateral views of WT and *smarca4^a50/a50 ^*retinas. There is no discernable signal in the retinas at 52 hpf while a retinal expression was observed in the public *in situ *hybridization data (ZFIN ID: ZDB-GENE-990603-12) that used this probe on embryos collected from high-pec to long-pec (~42-48 hpf) stages. Refer to Figure 1 for sample abbreviations. (B) The lateral view of a whole WT and *smarca4^a50/a50 ^*embryo at 36 and 52 hpf is shown to illustrate that the cb748 probe can detect signals in other embryonic regions as described in the public data. These include otic vesicle and myoseptum at 36 hpf, and otic vesicle and lower jaw at 52 hpf (black arrows). (C) The lateral view of the tail of a WT and *smarca4^a50/a50 ^*embryo to show the signal in the myoseptum (black arrow). Scale bar: 50 μm for (A & C), 100 μm for (B). Click here for file

Additional file 2 **Table S1. A summary of the microarray and the corresponding *in situ *hybridization results for the 27 Smarca4-regulated retinal genes with a positive *in situ *hybridization expression signal**. The fold changes of the four retinal comparisons: YR36/WR36, YR52/WR52, WR52/WR36 and YR52/YR36 from the original microarray analysis are shown here and used to generate the heatmap in Figure 1A. The significant comparisons are highlighted in bold-type font (See Methods and [12] for details). The same four retinal comparisons were conducted using the *in situ *hybridization samples. In these cases, the expression results were scored as over-expressed (1), no change (0) or under-expressed (-1). These observations are plotted in Figure 1B. If a significant microarray comparison does not have a corresponding change in the *in situ *hybridization or vice versa, the observation is highlighted by yellow in both microarray and *in situ *hybridization comparisons. The resulting identity matrix is plotted in Figure 1C. Click here for file

Additional file 3 **Table S2. A summary of the cellular expression patterns of the 25 Smarca4-regulated retinal genes**. The *in situ *hybridization signal of 25 genes was intense enough for discerning cellular expression domains unambiguously, which were scored as present (1) or absent (0) as defined in Figure 2A, B &2C. For 36 hpf ventral view: GC/BR - ganglion cell region or basal side of retina, OR-b - outer retina - basal, OR-a - outer retinal - apical. For 52 hpf ventral view: GC - ganglion cells, INL-b - inner nuclear layer - basal side, INL-m - inner nuclear layer - middle, INL-a - inner nuclear layer - apical side, ONL - outer nuclear layer. For 36 & 52 hpf lateral view: VP - ventral patch, AV - anterior ventral, AD - anterior dorsal, PD - posterior dorsal, PV - posterior ventral. The resulting matrix was used for the hierarchical clustering and generation of the heatmap in Figure 2D. Click here for file

Additional file 4 **Table S3. A summary of the cellular expression patterns of the three retinal-specific genes that are inferred to be independent of Smarca4 regulation**. Three genes were not inferred by the original factorial microarray analysis as Smarca4-regulated (see text for further discussion) and were inferred as genes that had a retinal-specific expression at both 36 and 52 hpf. Significance was defined by the *q-*value of the corresponding retinal specific expression contrast < 0.05 and the fold change > = 2 [12]. The fold changes of retina/whole embryo comparison at 36 and 52 hpf are shown here. The experimental results obtained from the *in situ *hybridization comparison were scored the same way as described in Figure 2. All corresponding *in situ *hybridization comparisons show the same qualitative change as in the microarray analysis, i.e. over-expression. Click here for file

Additional file 5 **Table S4. The PCR primers used for generation of riboprobes for *in situ *hybridization**. The primers were designed by Primer3 using the approach as described in the Materials and Methods section. The symbol, name, Affy ID, Genbank accession number of their corresponding genes are listed. The primer sequences and the size of the PCR product, as amplified by the specific pair of primers, are also shown here. Click here for file
