# Supplementary figures and images for: Cellular Expression of Smarca4 (Brg1)-regulated Genes in Zebrafish Retinas
Source: BMC Dev Biol. 2011 Jul 14;11:45. doi: 10.1186/1471-213X-11-45 (PMC3155967; doi:10.1186/1471-213X-11-45)

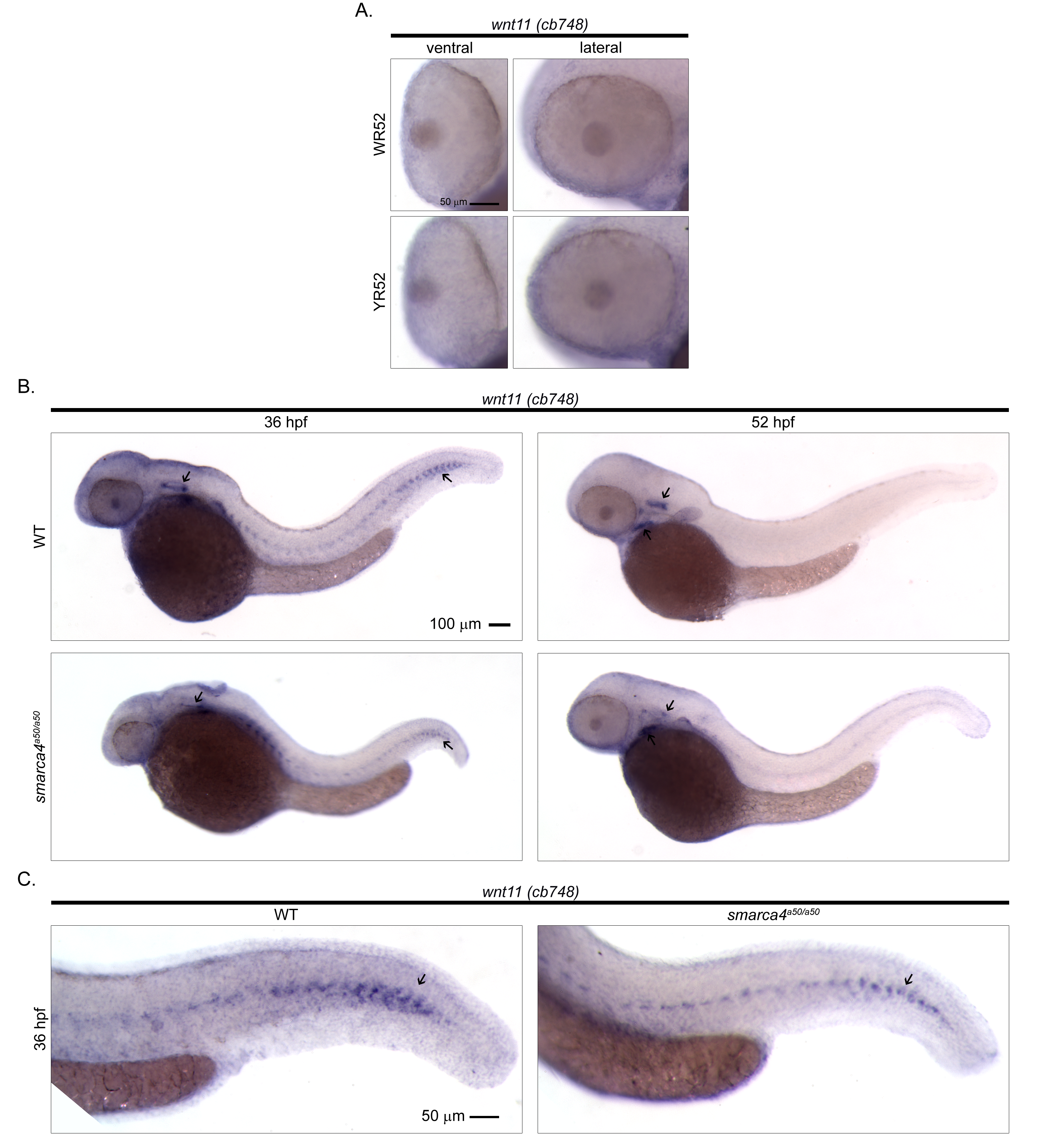

Supplement: Additional file 1 — Figure S1. Cellular expression pattern of wnt11 as identified by a previously reported riboprobe. The in situ hybridization experiment of wnt11 was repeated using a probe (cb748) that was obtained from ZIRC. (A) The ventral and the lateral views of WT and smarca4a50/a50 retinas. There is no discernable signal in the retinas at 52 hpf while a retinal expression was observed in the public in situ hybridization data (ZFIN ID: ZDB-GENE-990603-12) that used this probe on embryos collected from high-pec to long-pec (~42-48 hpf) stages. Refer to Figure 1 for sample abbreviations. (B) The lateral view of a whole WT and smarca4a50/a50 embryo at 36 and 52 hpf is shown to illustrate that the cb748 probe can detect signals in other embryonic regions as described in the public data. These include otic vesicle and myoseptum at 36 hpf, and otic vesicle and lower jaw at 52 hpf (black arrows). (C) The lateral view of the tail of a WT and smarca4a50/a50 embryo to show the signal in the myoseptum (black arrow). Scale bar: 50 μm for (A & C), 100 μm for (B). [file 1471-213X-11-45-S1.TIFF]
